# What Is the Cure for Neurasthenia?

**Published:** 1904-07-09

**Authors:** 


### WHAT IS THE CURE FOR NEURASTHENIA?

In an article contributed to the last volume of International Clinics, Dr. Robert T. Edes puts in a well-timed plea for the exercise of better discrimination in diagnosis and the use of more intelligent therapeutics than are commonly employed in dealing with neurasthenic patients. In the first place he makes a distinction between those cases where the condition is due to nervous exhaustion brought on by the stress and worry of modern existence, and those in which there is really a non-development of real strength due to a defective education or to an imagined weakness. The latter are not instances of nerve exhaustion at all, and unless this be fully appreciated both prognosis and treatment are likely to prove at fault. For the individual whose breakdown is due to overwork, the first essential therapeutic measure is bodily and mental rest. But it should be realised that this is a temporary expedient which in itself is insufficient to ensure that no recurrence of the illness will take place if the patient resumes his former habits of life. Something more than mere rest is necessary. Either the patient must resolve to so modify his future habits that his capacity for endurance will not be overtaxed, or he must undergo a course of training, so to speak, which will render him stronger and more fitted to bear the trials and worry of a strenuous life. Treatment, therefore, is resolved into two stages—a period of rest, during which the diet is regulated, any accessory complaints such as eye-strain, pelvic disorders, etc., attended to, and the patient's general condition improved. Then the muscles must be exercised. As Dr. Edes says, their active movement contributes not only to their own development but to the exercise of the motor centres, and, as soon as the movements are undertaken spontaneously, to that of the will and attention. The object, therefore, is to adopt exercises which will interest the patient, so that the will is having its proper motor outlet while the attention is directed not to the sensations and symptoms but to the accomplishment of some outside object. For instance, walking may be the best or the worst of exercises according to whether it is done with some pleasant object in view, or with an agreeable companion, or whether it is done only as a troublesome duty prescribed by the doctor. So with all sorts of exercises, employments, or games, the nature of the associations connected with them is of more consequence than the special muscles involved, and the moderate training of the attention in a healthy direction should be the object of chief importance.

